# *N*′-(3-Phenyl­allyl­idene)isonicotino­hydrazide

**DOI:** 10.1107/S1600536808026287

**Published:** 2008-08-20

**Authors:** Feng-Yu Bao

**Affiliations:** a Department of Applied Chemistry, College of Sciences, Henan Agricultural University, Zhengzhou 450002, People’s Republic of China

## Abstract

The asymmetric unit of the title compound, C_15_H_13_N_3_O, contains two similar mol­ecules. Each mol­ecule is non-planar, as indicated by the dihedral angles between the pyridine and benzene rings of 45.2 (2) and 56.6 (2)°. The crystal structure is consolidated by inter­molecular N—H⋯O hydrogen bonds.

## Related literature

For related literature, see: Kahwa *et al.* (1986 ▶); Qian *et al.* (2006 ▶); Santos *et al.* (2001 ▶).
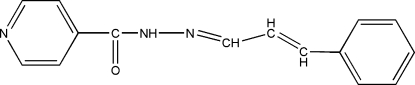


## Experimental

### 

#### Crystal data

C_15_H_13_N_3_O
*M* *_r_* = 251.28
Monoclinic, 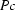

*a* = 12.608 (8) Å
*b* = 11.023 (7) Å
*c* = 10.044 (7) Å
β = 105.94 (3)°
*V* = 1342.2 (15) Å^3^
*Z* = 4
Mo *K*α radiation
μ = 0.08 mm^−1^
*T* = 291 (2) K
0.30 × 0.26 × 0.24 mm

#### Data collection

Bruker SMART APEX CCD area-detector diffractometer
Absorption correction: multi-scan (*SADABS*; Bruker, 2000 ▶) *T*_min_ = 0.98, *T*_max_ = 0.98
11645 measured reflections
3110 independent reflections
2784 reflections with *I* > 2σ(*I*)
*R*_int_ = 0.044

#### Refinement

*R*[*F* ^2^ > 2σ(*F* ^2^)] = 0.059
*wR*(*F* ^2^) = 0.134
*S* = 1.01
3110 reflections
349 parameters
2 restraints
H atoms treated by a mixture of independent and constrained refinement
Δρ_max_ = 0.20 e Å^−3^
Δρ_min_ = −0.29 e Å^−3^

### 

Data collection: *SMART* (Bruker, 2000 ▶); cell refinement: *SMART*; data reduction: *SAINT* (Bruker, 2000 ▶); program(s) used to solve structure: *SHELXTL* (Sheldrick, 2008 ▶); program(s) used to refine structure: *SHELXTL*; molecular graphics: *SHELXTL*; software used to prepare material for publication: *SHELXTL*.

## Supplementary Material

Crystal structure: contains datablocks global, I. DOI: 10.1107/S1600536808026287/ez2136sup1.cif

Structure factors: contains datablocks I. DOI: 10.1107/S1600536808026287/ez2136Isup2.hkl

Additional supplementary materials:  crystallographic information; 3D view; checkCIF report

## Figures and Tables

**Table 1:** Hydrogen-bond geometry (Å, °)

*D*—H⋯*A*	*D*—H	H⋯*A*	*D*⋯*A*	*D*—H⋯*A*
N2—H2*A*⋯O2^i^	0.86 (5)	2.19 (5)	3.050 (6)	174 (3)

Symmetry code: (i) 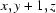.

**Fig. 1.**
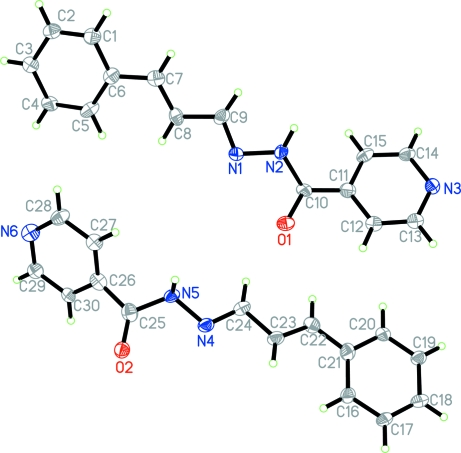
ORTEP plot of (I) showing the atom numbering scheme. Displacement ellipsoids are drawn at the 30% probability level.

## supplementary crystallographic information

### Comment

Interest in the chemistry of Schiff bases has increased considerably in recent years, mainly due to their novel properties and their application in the development of various proteins and enzymes (Kahwa *et al.*,  1986; Santos *et al.*, 2001). Structural information of Schiff base derivatives is useful in studying their coordination chemisty. As part of our research, we have synthesized the title compound (I) and report its crystal structure here.

The molecular structure is shown in Fig. 1. Each molecule is non-planar, with dihedral angles of 45.2 (2) and 56.6 (2)° between the pyridine ring and the benzene ring for the two molecules. Bond lengths and angles agree with those found for isonicotinohydrazide derivatives (Qian *et al.*, 2006).

Intermolecular N—H···O hydrogen bonds link pairs of molecules.

### Experimental

Pyridine-4-carboxylic acid hydrazide (1 mmol, 0.137 g) was dissolved in anhydrous methanol, whereafter H_2_SO_4_ (98%, 0.5 ml) was added and the mixture was stirred for several minutes at 351 K. A solution of cinnamaldehyde (1 mmol, 0.132 g) in methanol (8 ml) was then added dropwise and the mixture was stirred under reflux for 2 h. The product was isolated and recrystallized from dichloromethane, brown single crystals of (I) were obtained after 2 d.

### Refinement

H atoms on N2 and N5 were identified by difference Fourier map and refined isotropically. All other H atoms were placed in calculated positions, with C-H=0.93Å (aromatic), N-H = 0.96Å, and with U_iso_(H)=1.2U_eq_(C,N). In the absence of significant anomalous scattering effects, 2686 Friedel pairs have been merged.

### Crystal data

**Table:**

C_15_H_13_N_3_O	*F*_000_ = 528
*M**_r_* = 251.28	*D*_x_ = 1.244 Mg m^−^^3^
Monoclinic, *P**c*	Mo *K*α radiation λ = 0.71073 Å
Hall symbol: P -2yc	Cell parameters from 940 reflections
*a* = 12.608 (8) Å	θ = 2.5–20.5º
*b* = 11.023 (7) Å	µ = 0.08 mm^−^^1^
*c* = 10.044 (7) Å	*T* = 291 (2) K
β = 105.94 (3)º	Block, brown
*V* = 1342.2 (15) Å^3^	0.30 × 0.26 × 0.24 mm
*Z* = 4	

### Data collection

**Table:**

Bruker SMART APEX CCD area-detector diffractometer	3110 independent reflections
Radiation source: sealed tube	2784 reflections with *I* > 2σ(*I*)
Monochromator: graphite	*R*_int_ = 0.044
*T* = 291(2) K	θ_max_ = 27.7º
φ and ω scans	θ_min_ = 1.7º
Absorption correction: multi-scan(SADABS; Bruker, 2000)	*h* = −16→16
*T*_min_ = 0.98, *T*_max_ = 0.98	*k* = −13→14
11645 measured reflections	*l* = −13→12

### Refinement

**Table:**

Refinement on *F*^2^	Secondary atom site location: difference Fourier map
Least-squares matrix: full	Hydrogen site location: inferred from neighbouring sites
*R*[*F*^2^ > 2σ(*F*^2^)] = 0.059	H atoms treated by a mixture of independent and constrained refinement
*wR*(*F*^2^) = 0.134	*w* = 1/[σ^2^(*F*_o_^2^) + (0.05*P*)^2^ + 0.88*P*] where *P* = (*F*_o_^2^ + 2*F*_c_^2^)/3
*S* = 1.01	(Δ/σ)_max_ < 0.001
3110 reflections	Δρ_max_ = 0.20 e Å^−^^3^
349 parameters	Δρ_min_ = −0.29 e Å^−^^3^
2 restraints	Extinction correction: none
Primary atom site location: structure-invariant direct methods	

### Special details

**Table:**

Geometry. All e.s.d.'s (except the e.s.d. in the dihedral angle between two l.s. planes) are estimated using the full covariance matrix. The cell e.s.d.'s are taken into account individually in the estimation of e.s.d.'s in distances, angles and torsion angles; correlations between e.s.d.'s in cell parameters are only used when they are defined by crystal symmetry. An approximate (isotropic) treatment of cell e.s.d.'s is used for estimating e.s.d.'s involving l.s. planes.
Refinement. Refinement of *F*^2^ against ALL reflections. The weighted *R*-factor *wR* and goodness of fit *S* are based on *F*^2^, conventional *R*-factors *R* are based on *F*, with *F* set to zero for negative *F*^2^. The threshold expression of *F*^2^ > σ(*F*^2^) is used only for calculating *R*-factors(gt) *etc*. and is not relevant to the choice of reflections for refinement. *R*-factors based on *F*^2^ are statistically about twice as large as those based on *F*, and *R*- factors based on ALL data will be even larger.

### Fractional atomic coordinates and isotropic or equivalent isotropic displacement parameters (Å^2^)

**Table:**

	*x*	*y*	*z*	*U*_iso_*/*U*_eq_	
C1	1.2011 (4)	0.4816 (5)	0.8674 (5)	0.0472 (10)	
H1	1.2238	0.5562	0.8420	0.057*	
C2	1.2551 (4)	0.4303 (4)	0.9917 (5)	0.0457 (10)	
H2	1.3139	0.4716	1.0505	0.055*	
C3	1.2239 (4)	0.3174 (4)	1.0323 (5)	0.0417 (9)	
H3	1.2609	0.2838	1.1173	0.050*	
C4	1.1362 (4)	0.2566 (4)	0.9422 (4)	0.0418 (10)	
H4	1.1148	0.1809	0.9666	0.050*	
C5	1.0805 (4)	0.3081 (5)	0.8167 (4)	0.0469 (11)	
H5	1.0219	0.2672	0.7571	0.056*	
C6	1.1126 (3)	0.4217 (4)	0.7798 (5)	0.0421 (9)	
C7	1.0508 (3)	0.4875 (4)	0.6585 (5)	0.0426 (10)	
H7	1.0694	0.5672	0.6446	0.051*	
C8	0.9646 (4)	0.4321 (4)	0.5637 (5)	0.0436 (10)	
H8	0.9451	0.3521	0.5747	0.052*	
C9	0.9049 (4)	0.5050 (4)	0.4446 (4)	0.0435 (10)	
H9	0.9175	0.5870	0.4333	0.052*	
C10	0.6810 (4)	0.4686 (4)	0.1451 (4)	0.0391 (9)	
C11	0.5998 (4)	0.5425 (4)	0.0516 (4)	0.0438 (10)	
C12	0.5471 (3)	0.5017 (4)	−0.0823 (4)	0.0356 (8)	
H12	0.5587	0.4233	−0.1093	0.043*	
C13	0.4793 (4)	0.5778 (4)	−0.1716 (5)	0.0413 (9)	
H13	0.4438	0.5497	−0.2599	0.050*	
C14	0.5136 (4)	0.7372 (4)	−0.0082 (4)	0.0427 (9)	
H14	0.5025	0.8165	0.0165	0.051*	
C15	0.5845 (3)	0.6620 (4)	0.0872 (4)	0.0432 (10)	
H15	0.6216	0.6914	0.1743	0.052*	
C16	0.4052 (4)	0.0511 (4)	−0.2985 (4)	0.0424 (9)	
H16	0.4482	−0.0181	−0.2728	0.051*	
C17	0.3238 (3)	0.0544 (4)	−0.4232 (4)	0.0400 (9)	
H17	0.3127	−0.0128	−0.4814	0.048*	
C18	0.2588 (4)	0.1560 (4)	−0.4623 (5)	0.0455 (10)	
H18	0.2039	0.1569	−0.5459	0.055*	
C19	0.2761 (4)	0.2580 (4)	−0.3752 (4)	0.0417 (9)	
H19	0.2328	0.3269	−0.4015	0.050*	
C20	0.3577 (3)	0.2565 (4)	−0.2498 (4)	0.0356 (8)	
H20	0.3689	0.3242	−0.1923	0.043*	
C21	0.4226 (4)	0.1536 (4)	−0.2104 (4)	0.0462 (10)	
C22	0.5026 (4)	0.1586 (4)	−0.0775 (5)	0.0462 (10)	
H22	0.5019	0.2227	−0.0175	0.055*	
C23	0.5807 (4)	0.0677 (4)	−0.0391 (4)	0.0433 (10)	
H23	0.5931	0.0067	−0.0970	0.052*	
C24	0.6411 (3)	0.0828 (4)	0.1081 (4)	0.0398 (9)	
H24	0.6423	0.1533	0.1594	0.048*	
C25	0.8300 (4)	−0.1108 (5)	0.3489 (5)	0.0502 (11)	
C26	0.8954 (4)	−0.0909 (4)	0.4904 (5)	0.0441 (10)	
C27	0.9750 (4)	−0.0014 (4)	0.5288 (5)	0.0405 (9)	
H27	0.9822	0.0570	0.4650	0.049*	
C28	1.0442 (4)	0.0012 (5)	0.6632 (5)	0.0505 (11)	
H28	1.0965	0.0623	0.6904	0.061*	
C29	0.9555 (3)	−0.1774 (4)	0.7183 (4)	0.0421 (9)	
H29	0.9483	−0.2360	0.7819	0.051*	
C30	0.8865 (3)	−0.1796 (3)	0.5848 (4)	0.0337 (8)	
H30	0.8341	−0.2407	0.5582	0.040*	
N1	0.8334 (3)	0.4414 (3)	0.3586 (4)	0.0397 (8)	
N2	0.7663 (3)	0.5206 (4)	0.2548 (4)	0.0423 (9)	
H2A	0.777 (4)	0.598 (5)	0.259 (5)	0.051*	
N3	0.4603 (3)	0.6964 (3)	−0.1372 (3)	0.0392 (8)	
N4	0.6918 (3)	−0.0162 (3)	0.1548 (4)	0.0424 (8)	
N5	0.7800 (3)	−0.0023 (3)	0.2797 (3)	0.0368 (8)	
H5A	0.833 (4)	0.042 (4)	0.259 (5)	0.044*	
N6	1.0342 (3)	−0.0891 (4)	0.7569 (4)	0.0491 (9)	
O1	0.6774 (2)	0.3578 (3)	0.1322 (3)	0.0434 (7)	
O2	0.8172 (2)	−0.2090 (3)	0.2914 (3)	0.0440 (7)	

### Atomic displacement parameters (Å^2^)

**Table:**

	*U*^11^	*U*^22^	*U*^33^	*U*^12^	*U*^13^	*U*^23^
C1	0.042 (2)	0.052 (3)	0.051 (3)	−0.006 (2)	0.019 (2)	0.004 (2)
C2	0.042 (2)	0.048 (2)	0.049 (3)	−0.0034 (19)	0.017 (2)	−0.011 (2)
C3	0.043 (2)	0.044 (2)	0.043 (2)	0.0020 (18)	0.0214 (19)	−0.0022 (18)
C4	0.048 (2)	0.042 (2)	0.042 (2)	−0.0146 (18)	0.0227 (19)	0.0100 (17)
C5	0.037 (2)	0.071 (3)	0.035 (2)	−0.014 (2)	0.0134 (17)	−0.010 (2)
C6	0.0332 (18)	0.050 (2)	0.046 (2)	0.0070 (18)	0.0149 (17)	−0.0088 (19)
C7	0.037 (2)	0.043 (2)	0.054 (3)	0.0071 (18)	0.0238 (19)	−0.014 (2)
C8	0.040 (2)	0.041 (2)	0.053 (3)	0.0142 (18)	0.0167 (19)	0.0057 (19)
C9	0.051 (2)	0.050 (2)	0.0264 (19)	0.003 (2)	0.0053 (17)	−0.0123 (18)
C10	0.044 (2)	0.036 (2)	0.040 (2)	−0.0233 (18)	0.0151 (17)	0.0063 (17)
C11	0.054 (3)	0.041 (2)	0.038 (2)	−0.006 (2)	0.0163 (19)	−0.0013 (18)
C12	0.0331 (19)	0.038 (2)	0.043 (2)	0.0074 (16)	0.0221 (16)	−0.0088 (16)
C13	0.042 (2)	0.046 (2)	0.040 (2)	−0.0041 (19)	0.0172 (18)	−0.0042 (18)
C14	0.048 (2)	0.042 (2)	0.037 (2)	−0.0053 (19)	0.0103 (18)	−0.0103 (18)
C15	0.041 (2)	0.056 (3)	0.032 (2)	−0.0076 (19)	0.0092 (17)	−0.0133 (18)
C16	0.051 (2)	0.045 (2)	0.031 (2)	−0.005 (2)	0.0121 (17)	0.0038 (17)
C17	0.036 (2)	0.046 (2)	0.036 (2)	−0.0121 (17)	0.0074 (17)	−0.0079 (17)
C18	0.047 (2)	0.047 (2)	0.043 (2)	−0.0120 (19)	0.0108 (19)	−0.013 (2)
C19	0.049 (2)	0.043 (2)	0.034 (2)	−0.0036 (19)	0.0128 (18)	−0.0051 (17)
C20	0.0386 (19)	0.036 (2)	0.037 (2)	−0.0144 (16)	0.0175 (16)	0.0037 (16)
C21	0.055 (3)	0.051 (3)	0.037 (2)	−0.004 (2)	0.0198 (19)	0.0099 (19)
C22	0.046 (2)	0.044 (2)	0.051 (3)	0.0147 (19)	0.018 (2)	−0.0061 (19)
C23	0.044 (2)	0.045 (2)	0.040 (2)	0.0018 (19)	0.0095 (18)	−0.0135 (18)
C24	0.0314 (18)	0.052 (2)	0.037 (2)	−0.0043 (17)	0.0113 (16)	−0.0110 (18)
C25	0.050 (2)	0.052 (3)	0.054 (3)	0.013 (2)	0.022 (2)	0.003 (2)
C26	0.047 (2)	0.045 (2)	0.049 (3)	0.003 (2)	0.028 (2)	0.005 (2)
C27	0.038 (2)	0.046 (2)	0.044 (2)	0.0001 (17)	0.0225 (18)	0.0012 (18)
C28	0.050 (2)	0.063 (3)	0.038 (2)	−0.011 (2)	0.0126 (19)	−0.009 (2)
C29	0.040 (2)	0.047 (2)	0.044 (2)	0.0067 (18)	0.0202 (18)	−0.0077 (18)
C30	0.0285 (16)	0.0339 (19)	0.044 (2)	0.0102 (14)	0.0190 (15)	−0.0027 (16)
N1	0.0374 (17)	0.0327 (17)	0.0451 (19)	−0.0052 (14)	0.0045 (14)	0.0001 (14)
N2	0.0305 (17)	0.045 (2)	0.0435 (19)	−0.0151 (15)	−0.0034 (14)	0.0138 (16)
N3	0.0488 (19)	0.0410 (19)	0.0321 (17)	0.0031 (16)	0.0182 (14)	0.0002 (15)
N4	0.055 (2)	0.0406 (19)	0.0303 (17)	−0.0081 (17)	0.0093 (16)	−0.0039 (15)
N5	0.0338 (17)	0.0399 (18)	0.0350 (18)	−0.0151 (14)	0.0064 (14)	−0.0011 (14)
N6	0.043 (2)	0.062 (2)	0.049 (2)	−0.0020 (18)	0.0230 (17)	−0.0041 (19)
O1	0.0396 (15)	0.0449 (17)	0.0474 (17)	−0.0096 (13)	0.0152 (13)	−0.0059 (13)
O2	0.0434 (15)	0.0455 (17)	0.0413 (16)	0.0001 (13)	0.0084 (12)	0.0034 (14)

### Geometric parameters (Å, °)

**Table:**

C1—C2	1.369 (7)	C16—H16	0.9300
C1—C6	1.385 (7)	C17—C18	1.379 (7)
C1—H1	0.9300	C17—H17	0.9300
C2—C3	1.400 (6)	C18—C19	1.405 (6)
C2—H2	0.9300	C18—H18	0.9300
C3—C4	1.394 (6)	C19—C20	1.391 (6)
C3—H3	0.9300	C19—H19	0.9300
C4—C5	1.386 (6)	C20—C21	1.391 (6)
C4—H4	0.9300	C20—H20	0.9300
C5—C6	1.397 (7)	C21—C22	1.437 (6)
C5—H5	0.9300	C22—C23	1.383 (6)
C6—C7	1.447 (7)	C22—H22	0.9300
C7—C8	1.375 (7)	C23—C24	1.475 (6)
C7—H7	0.9300	C23—H23	0.9300
C8—C9	1.467 (6)	C24—N4	1.287 (6)
C8—H8	0.9300	C24—H24	0.9300
C9—N1	1.273 (5)	C25—O2	1.216 (6)
C9—H9	0.9300	C25—N5	1.439 (6)
C10—O1	1.228 (5)	C25—C26	1.451 (7)
C10—N2	1.432 (5)	C26—C27	1.385 (6)
C10—C11	1.437 (6)	C26—C30	1.388 (6)
C11—C15	1.393 (6)	C27—C28	1.391 (7)
C11—C12	1.400 (6)	C27—H27	0.9300
C12—C13	1.347 (6)	C28—N6	1.399 (7)
C12—H12	0.9300	C28—H28	0.9300
C13—N3	1.390 (6)	C29—N6	1.368 (6)
C13—H13	0.9300	C29—C30	1.384 (6)
C14—N3	1.362 (5)	C29—H29	0.9300
C14—C15	1.390 (7)	C30—H30	0.9300
C14—H14	0.9300	N1—N2	1.442 (5)
C15—H15	0.9300	N2—H2A	0.86 (5)
C16—C17	1.385 (6)	N4—N5	1.437 (5)
C16—C21	1.415 (7)	N5—H5A	0.90 (5)
			
C2—C1—C6	119.7 (5)	C16—C17—H17	119.6
C2—C1—H1	120.1	C17—C18—C19	119.6 (4)
C6—C1—H1	120.1	C17—C18—H18	120.2
C1—C2—C3	121.6 (4)	C19—C18—H18	120.2
C1—C2—H2	119.2	C20—C19—C18	120.3 (4)
C3—C2—H2	119.2	C20—C19—H19	119.9
C4—C3—C2	118.4 (4)	C18—C19—H19	119.9
C4—C3—H3	120.8	C21—C20—C19	119.9 (4)
C2—C3—H3	120.8	C21—C20—H20	120.1
C5—C4—C3	120.5 (4)	C19—C20—H20	120.1
C5—C4—H4	119.8	C20—C21—C16	119.7 (4)
C3—C4—H4	119.8	C20—C21—C22	116.2 (4)
C4—C5—C6	119.9 (4)	C16—C21—C22	124.1 (4)
C4—C5—H5	120.1	C23—C22—C21	119.7 (4)
C6—C5—H5	120.1	C23—C22—H22	120.1
C1—C6—C5	120.0 (4)	C21—C22—H22	120.1
C1—C6—C7	116.7 (5)	C22—C23—C24	109.2 (4)
C5—C6—C7	123.0 (4)	C22—C23—H23	125.4
C8—C7—C6	120.0 (4)	C24—C23—H23	125.4
C8—C7—H7	120.0	N4—C24—C23	109.7 (4)
C6—C7—H7	120.0	N4—C24—H24	125.1
C7—C8—C9	116.9 (4)	C23—C24—H24	125.1
C7—C8—H8	121.5	O2—C25—N5	121.8 (5)
C9—C8—H8	121.5	O2—C25—C26	124.4 (4)
N1—C9—C8	111.1 (4)	N5—C25—C26	113.8 (4)
N1—C9—H9	124.4	C27—C26—C30	120.0 (4)
C8—C9—H9	124.4	C27—C26—C25	123.7 (4)
O1—C10—N2	118.5 (4)	C30—C26—C25	115.6 (4)
O1—C10—C11	119.7 (4)	C26—C27—C28	119.9 (4)
N2—C10—C11	121.7 (4)	C26—C27—H27	120.1
C15—C11—C12	119.1 (4)	C28—C27—H27	120.1
C15—C11—C10	119.5 (4)	C27—C28—N6	119.3 (4)
C12—C11—C10	120.7 (4)	C27—C28—H28	120.3
C13—C12—C11	119.1 (4)	N6—C28—H28	120.3
C13—C12—H12	120.4	N6—C29—C30	120.1 (4)
C11—C12—H12	120.4	N6—C29—H29	120.0
C12—C13—N3	122.8 (4)	C30—C29—H29	120.0
C12—C13—H13	118.6	C29—C30—C26	120.2 (4)
N3—C13—H13	118.6	C29—C30—H30	119.9
N3—C14—C15	121.0 (4)	C26—C30—H30	119.9
N3—C14—H14	119.5	C9—N1—N2	108.8 (4)
C15—C14—H14	119.5	C10—N2—N1	118.8 (3)
C14—C15—C11	119.7 (4)	C10—N2—H2A	121 (3)
C14—C15—H15	120.1	N1—N2—H2A	121 (3)
C11—C15—H15	120.1	C14—N3—C13	118.2 (4)
C17—C16—C21	119.6 (4)	C24—N4—N5	114.5 (3)
C17—C16—H16	120.2	N4—N5—C25	117.7 (4)
C21—C16—H16	120.2	N4—N5—H5A	108 (3)
C18—C17—C16	120.9 (4)	C25—N5—H5A	108 (3)
C18—C17—H17	119.6	C29—N6—C28	120.5 (4)

### Hydrogen-bond geometry (Å, °)

**Table:**

*D*—H···*A*	*D*—H	H···*A*	*D*···*A*	*D*—H···*A*
N2—H2A···O2^i^	0.86 (5)	2.19 (5)	3.050 (6)	174 (3)

Symmetry codes: (i) *x*, *y*+1, *z*.
